# Subdiaphragmatic activity-related artifacts in myocardial perfusion scintigraphy

**DOI:** 10.2478/raon-2024-0053

**Published:** 2024-09-15

**Authors:** Anja Strok, Barbara Guzic Salobir, Monika Stalc, Katja Zaletel

**Affiliations:** Division of Nuclear Medicine, University Medical Centre Ljubljana, Ljubljana, Slovenia; Faculty of Medicine, University of Ljubljana, Ljubljana, Slovenia

**Keywords:** myocardial perfusion imaging, artifacts, subdiaphragmatic activity, single photon emission computed tomography, intervention

## Abstract

**Background:**

Myocardial perfusion imaging (MPI) with single photon emission computed tomography is an established non-invasive technique for assessing myocardial ischemia. This method involves the intravenous administration of a radiopharmaceutical that accumulates in the heart muscle proportional to regional blood flow. However, image quality and diagnostic accuracy can be compromised by various technical and patient-related factors, including high non-specific radiopharmaceutical uptake in abdominal organs such as the stomach, intestines, liver, and gall-bladder, leading to subdiaphragmatic artifacts. These artifacts are particularly problematic for evaluating inferior wall perfusion and often necessitate repeated imaging, which decreases gamma camera availability and prolongs imaging times.

**Conclusions:**

Despite numerous investigated techniques to reduce interfering gastrointestinal activity, results have been inconsistent, and current MPI guidelines provide scant information on effective procedures to mitigate this issue. Based on our experience, some possible approaches to reducing artifacts include choosing stress testing with an exercise stress test, when possible, late imaging, fluid intake, and consuming carbonated water immediately before imaging.

## Introduction

Myocardial perfusion imaging (MPI) with single photon emission computer tomography (SPECT) is an established non-invasive imaging modality for evaluating myocardial ischemia. It is based on the intravenous application of a radiopharmaceutical that accumulates in the heart muscle in proportion to the regional blood flow.^1,2^

The radioactive isotope emits energy in the form of gamma rays (photons), which are detected by a tomography gamma camera approximately one hour after the application of the radiopharmaceutical. The data on the intensity of the accumulation of the radiopharmaceutical in the heart muscle is converted into images by the computer. Thus, scintigrams are generated to assess blood flow to the heart muscle during stress (exercise or pharmacological) and at rest.^1,2^

The quality of the visual and quantitative analysis of MPI images is influenced by many technical and patient-related factors.^3,4^ Some limitations arise directly from the characteristics of the radiopharmaceuticals. They exhibit high unspecific uptake in the abdominal organs (stomach, intestines, liver, and gallbladder), which can create artifacts.^1,2,3^ Subdiaphragmatic activity is one of the most prevalent artifacts in SPECT imaging^3,4^, often interfering with the evaluation of inferior wall perfusion in approximately 10–50% of cases.^5^

Artifacts in the inferior myocardial wall caused by subdiaphragmatic activity present a significant challenge by diminishing image quality and the diagnostic accuracy of the study. This can lead to suboptimal or even inadequate patient management.^3^ In daily practice, abdominal activity interference often necessitates repeated imaging for some patients, which limits gamma camera availability, prolongs imaging times, and increases radiation exposure for both personnel and patients in the waiting room.

Currently, there is no standard approach to determine which technique(s) are most effective in reducing interfering subdiaphragmatic activity. Multiple techniques have been investigated to reduce interfering gastrointestinal activity while waiting for imaging, but results have been inconsistent. In MPI guidelines, information about procedures to reduce intestinal and liver activity is scarce.

In this review, we describe the effect of different interventions on subdiaphragmatic activity in MPI.

## Subdiaphragmatic artifacts on myocardial perfusion imaging

The radiopharmaceutical used for MPI consists of a radioactive isotope of tehnecium-99m (^99m^Tc) bound to a tetrofosmin or sestamibi molecule, which binds irreversibly to viable myocytes. In addition to the heart, radiopharmaceuticals used for MPI also accumulate in other organs and tissues. Initially, they localize in the liver due to their high lipophilicity and are then excreted via the hepatobiliary system into the duodenum. From there, tracer activity will move distally in the small bowel, or it may reflux into the stomach. Radiotracer can also accumulate in the gastric mucosa by active transport and retention in mitochondria or as a result of the dissociation of a tracer molecule and uptake of free ^99m^Tc-pertechnetate.^3,6,7^ This subdiaphragmatic radioactivity accumulation in the immediate vicinity of the heart causes artifacts, which interfere with the correct assessment of perfusion in the heart muscle, most often in the inferior wall of the left ventricle. In rare cases, such as with a hiatal hernia, the lateral wall can also be affected.^3^

Artifacts can cause either apparent increased or decreased activity in the adjacent inferior wall of the left ventricle, leading to false-negative or false-positive inferior wall perfusion defects, thereby mimicking ischemia, or concealing true perfusion defects.^3,4,8^

Scatter radiation from the radiotracer, combined with the effect of volume averaging, can create the appearance of increased perfusion in the inferior myocardial wall, which might mask a true perfusion defect in this region. On the other hand, scattering or superimposition of subdiaphragmatic activity on the inferior myocardial wall may cause normalization problems throughout the remainder of the left ventricle, resulting in the appearance of relatively low activity and simulating an extensive perfusion defect.^3,4^

**FIGURE 1.**
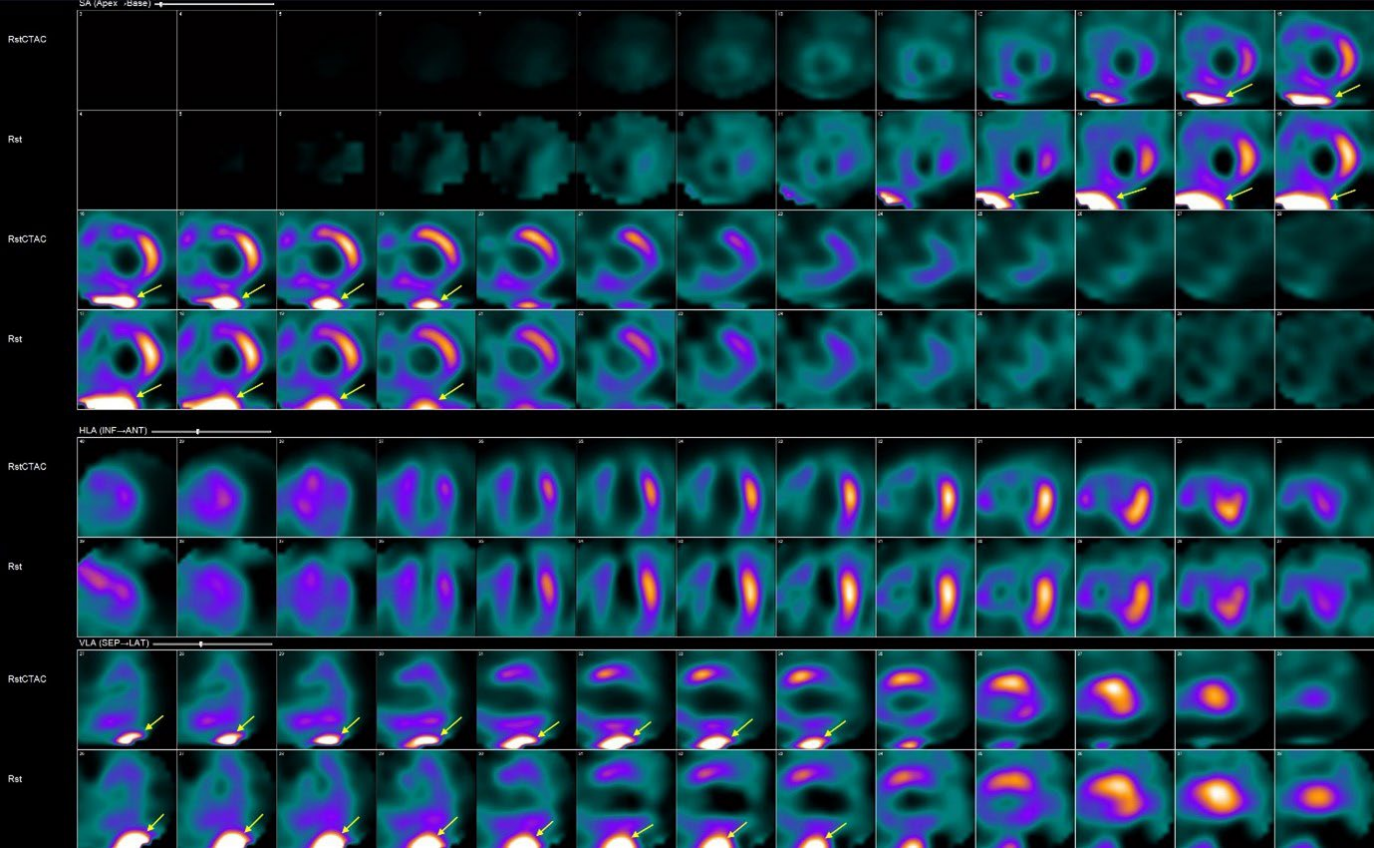
Interfering subdiaphragmatic activity (yellow arrow) on short and vertical long axis images of the left ventricle with (upper raw) and without attenuation correction (lower raw).

In clinical practice, the most common way of evaluating interfering extracardiac artifacts is visually on reconstructed SPECT images (Figure 1).

When the interfering activity could result in either a significant overestimation or underestimation of uptake in the myocardium, scans are normally repeated after 0.5–1 hour.

For study purposes, the intensity of interfering artifacts can be visually scored using a grading scale, similar to the one used previously by Albuitahi *et al*.^9^ and Bresser *et al*.^8^. The grading scale ranges from 0 to 3: 0 for absent subdiaphragmatic activity, 1 for mild subdiaphragmatic activity with no impact on visual interpretation, 2 for moderate subdiaphragmatic activity with a significant effect on interpretation, and 3 for severe subdiaphragmatic activity leading to a substantial impact on interpretation. Moderate and severe subdiaphragmatic tracer activity is considered relevant for the interpretation of MPI scans.

**FIGURE 2.**
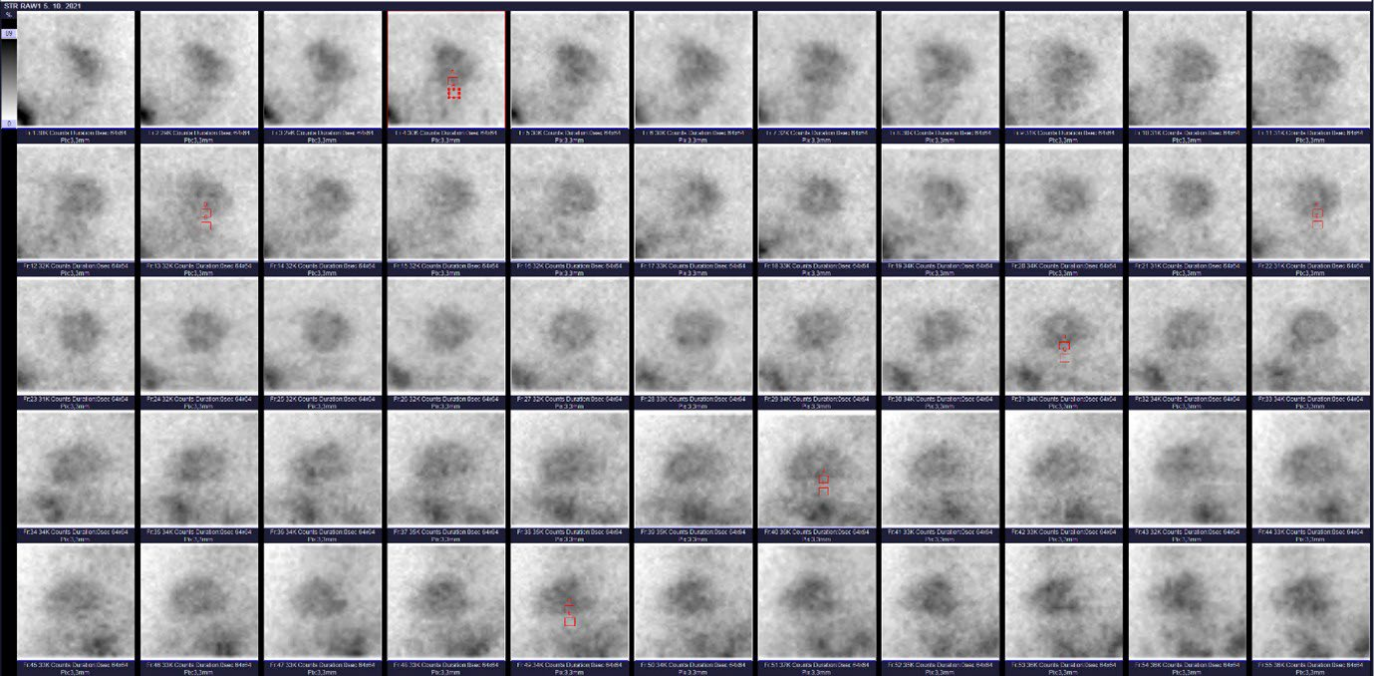
An example of manually drawn regions of interests (ROIs) over the midportion of the inferior wall of the myocardium and the adjacent underlying abdominal regions on multiple projections on raw planar images of the stress study. After pixel counts were obtained for every ROI, the mean myocardial and extracardiac counts were used to calculate the ratio of myocardial to extracardiac activity (MYO:EXT ratio).

Artifacts can also be analyzed semi quantitatively on raw planar scintigrams by calculating the ratio between the myocardial and extracardiac activity (MYO:EXT ratio), a metric that has been well-studied and validated in multiple previous investigations.^8,10,11,12,13,14^ The MYO:EXT ratio compares the inferior wall of the left ventricle myocardium to the infra-cardiac region^10,11^ and correlates strongly with the level of activity interfering with image interpretation or, as mentioned before, visual grading.^10^ Because this method is time-consuming, it is mainly used for academic purposes and not in routine clinical practice. The method of obtaining this ratio is shown in Figure 2.

## Type of stress testing and subdiaphragmatic artefacts

The frequency and intensity of the interfering extracardiac activity are affected by the patient’s physiological characteristics as well as the type of test performed. It is well-known that subdiaphragmatic activity is higher after vasodilator stress testing compared to exercise stress due to increased hepatic and gastrointestinal blood flow.^1,7,10^

Therefore, whenever the patient can achieve a sufficient level of exercise and has no contraindications, a stress study on the bicycle or treadmill is a preferred method. In patients who are unable to exercise or achieve sufficient exercise workload, or who have complete left bundle branch block or a predominantly electro systolic rhythm with a pacemaker implanted, pharmacological stress with vasodilators is used. Regadenosone is the most commonly used pharmacologic agent for stress testing in many centres, while dipyridamole and adenosine are less common.^1,2^

Vasodilators induce myocardial hyperaemia interacting with adenosine receptors. Regadenosone is a highly specific adenosine A2A receptor agonist that induces coronary vasodilation. Adenosine and dipyridamole are less specific agents and they also stimulate the A1, A2B, and A3 adenosine receptors.^1,4^ For that reason and because only about 5% of the cardiac output goes into the myocardial vasculature and the majority of the radiopharmaceutical distributes into other organs and tissues^7^, vasodilators provoke known and unwanted adverse effects^1^, such as depressed activity and conduction of the sinoatrial and atrioventricular nodes and possible atrioventricular heart block (receptor A1), peripheral vasodilation and hypotension (receptor A2B), and bronchoconstriction which can lead to severe and life-threatening events (receptor A3).^2,4^ By promoting peripheral vasodilatation and dilatation of the splanchnic vasculature, pharmacological stress test leads to more pronounced accumulation of radiopharmaceuticals in the abdominal organs compared to exercise stress.^1,3,4,10^

Due to established facts, the addition of low-level exercise along with the vasodilator stress is recommended practice in guidelines. It minimizes artifacts by increasing skeletal muscle blood flow and reducing splanchnic blood flow to the viscera.^1,2,4,15,16^ Combining the two methods results in improved image quality.^2^ Additionally, the combination of a vasodilator with a low-level exercise protocol tailored to the abilities of the individual patient helps significantly reduce vasodilator-induced side effects (flushing, dizziness, nausea, headache, hypotension).^1^

Based on our experience (unpublished data), approximately 60% of the patients require a pharmacological stress test, and even 80% of the subjects during the COVID-19 epidemic in the years 2020–2022.

The use of pharmacologic stress testing has increased due to factors such as poor physical performance, general frailty and musculoskeletal conditions that hinder walking.^17^ Furthermore, in response to the COVID-19 pandemic, the American Society of Nuclear Cardiology and the Society of Nuclear Medicine and Molecular Imaging issued recommendations aimed at reducing viral exposure risk for healthcare workers and patients. These guidelines highlight the importance of minimizing interaction time and maximizing physical distancing. To reduce droplet exposure to exercise staff and limit close contact, pharmacological stress protocols using vasodilator agents have been preferred.^18^

## Technical aspects of minimizing subdiaphragmatic artifacts

Subdiaphragmatic artifacts can be reduced to some extent by the usage of iterative reconstruction methods during processing or attenuation correction.^1,19^

Attenuation correction methods can be based on traditional line-source transmission or CT-based with attenuation maps in novel SPECT/CT systems and are most useful for correcting artifacts due to soft tissue attenuation (originating from the breast or left hemidiaphragm). While a substantial number of artifacts can be corrected with these methods, but a few may also arise with their use.^4^ Scatter effects may be more pronounced on attenuation-corrected images.^19^

The use of iterative reconstruction methods has been recommended to minimize artifacts related to extracardiac activity. The iterative process is well suitable to include physical effects, such as photon attenuation and contributions from photons scattered in the patient. Because iterative techniques have been demonstrated to produce superior image reconstructions, they are preferred over traditional filtered-back projections (FBP).^1^ Prominent infracardiac activity can result in apparent decreased activity in the adjacent myocardium due to the reconstruction algorithm used in FBP. This is because relatively large streaks of negative numbers due to the ramp filter pass through the heart region and thus reduce the counts.^1,3^ This leads to artefactual decreased activity adjacent to hot objects. The phenomenon worsens with greater the subdiaphragmatic activity.^3^

Cardiac perfusion images are usually obtained with the patient in the supine position. Studies have demonstrated that in the presence of an inferior wall artifact in the stress supine MPI, a positional change (prone imaging) can be an effective technique to eliminate common artifacts. Altering the standard patient position can help overcome not only attenuation artifacts but also interfering with external activity by lowering of the diaphragm and displacing abdominal organs away from the inferior myocardial wall.^1,4,20^

In a few patients, duodenogastric reflux is observed, which can affect the quality of the scans. To alleviate the refluxed activity, some suggest positioning the patients lying on their right-hand side for 20 minutes.^10^

## Impact of food and liquid intake

The most common techniques used to reduce interfering activity involve the administration of food or liquids between injection and imaging. This is done to either stimulate hepatobiliary clearance of tracer or to distend the stomach and push the bowel loops inferiorly, further from the myocardium (volume effect). Techniques such as consuming a fatty meal, solid food, milk, lemon juice, carbonated beverages, plain water, or combined interventions have been explored with variable results^6,8,10,11,12,13,14,19,21,22^, and currently, there is no standard approach to determining which technique is most effective in reducing the artifacts.^1^

**FIGURE 3.**
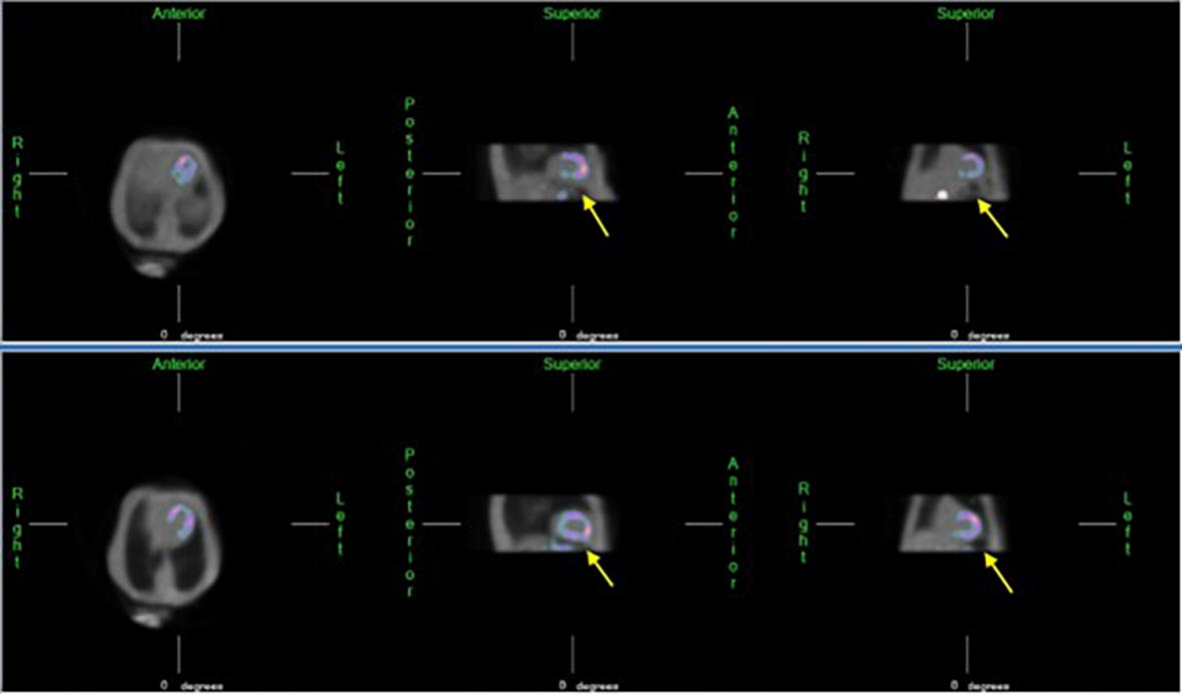
By administering carbonated water before imaging, carbon dioxide gas may additionally expand the stomach. The upper part of the stomach adjacent to the inferior wall of the heart is mainly filled with gas instead of water. By distending the stomach, we increase the distance between the gut and the heart, thereby reducing imaging artifacts (yellow arrow).

A recently published randomized study did not confirm the significant effectiveness of concomitant drinking of lemonade and carbonated mineral water in reducing subdiaphragmatic activity.^8^ On contrary, one of the previous studies showed a significant reduction in subdiaphragmatic activity by using smaller amounts of carbonated beverages. The study protocol was advantageous for patients with heart or renal failure in whom the intake of water may be a problem. Because it is desirable for these patients to drink as little water as possible and given that small volumes of soda water induce a greater volume expansion of the stomach compared to normal water, the use of carbonated beverages is the preferred method.^11^ Also, Hussain *et al*. demonstrated that ingestion of carbonated water significantly improved an interfering gut artifact in the majority of their patients.^14^ An example of the effect of carbonated water is shown on Figure 3.

Peace and colleagues found no significant effect of drinking water and fat milk^10^, while others observed a reduced accumulation of radiopharmaceuticals in the gastrointestinal tract when combined with drinking milk and water. They hypothesized that milk stimulates liver clearance and peristaltic movement, while water reduces activity in the stomach and accelerates the transition of biliary-excreted activity along the bowel tract.^19^

Similarly, in a study examining the impact of multiple techniques (drinking lemon juice, water, milk or a combination of measures), milk in-take proved to be an important factor in reducing subdiaphragmatic activity, likely due in part to faster gallbladder drainage after ingesting a fatty meal.^6^ They also found that the quality of scintigrams improved when drinking fluids and eating food simultaneously.^21^ This is in agreement with the results of an earlier study by Boz *et al*., where the volume effect after consuming water and a sandwich was investigated, and the usefulness of stomach fullness on extracardiac activity was demonstrated by comparing patients in fasting and non-fasting states. They increased the volume of the stomach with a combination of fluids and solid food to push the intestine caudally and thus remove intestinal artifacts further away from the myocardium.^12^

## Impact of drugs

The use of drugs that stimulate hepatobiliary clearance or gastric motility, such as erythromycin and metoclopramide, has been reviewed with varying results.^7,10^ Metoclopramide, used for treating and preventing nausea and vomiting, is known for accelerating intestinal transit but had no effect on abdominal activity in MPI and was consequently not recommended for routine practice.^7,23^

Drugs like the antibiotic erythromycin, which mimics motilin and leads to faster gastric emptying, have yielded favourable results but have been studied only in small groups.^7^

Some have hypothesized that iodinated oral contrast could be used to absorb gamma rays emitted from bowel activity but came to conclusion that some reduction in infracardiac activity was probably due to the volume effect.^10,24^

It was reported that proton-pump inhibitors, used to reduce stomach acid production, increase the accumulation of radiopharmaceutical in the stomach wall and can jeopardize the quality of MPI scans.^7^

None of these interventions and data seem to have become an integral part of the imaging protocol, and more recent and comprehensive studies with a larger number of patients are needed.

## The impact of fasting

Gastrointestinal activity and related artifacts were less pronounced in patients who were fasting or had only eaten a light meal prior to examination.^3^

## The role of late scanning

One of the most important approaches to minimize interfering subdiaphragmatic activity is to wait an adequate amount of time between radiopharmaceutical administration and imaging.^3^

Because MPI radiopharmaceuticals are cleared from the liver at a greater rate than from the heart, delaying the imaging allows physiologic clearance of the tracer from regions adjacent to the heart and has the benefit of reducing interfering hepatic activity.^1,2,4,9,10,21^

The recommended time interval between injection and acquisition for Tc99m- tetrofosmin by the manufacturer to yield the best image quality in relation to subdiaphragmatic activity is as soon as 15 minutes. However, MPI guidelines advise later post-injection acquisitions. Minimum delays of approximately 15 minutes for exercise, 30 to 45 minutes for rest, and 45 to 60 minutes for pharmacologic stress studies are optimal. Longer delays for repeated studies (up to 2 hours) can be used when needed.^1,2^

The prolongation of the time between radiopharmaceutical application and imaging improves the quality of scintigrams^4,8,10^, but some authors, on the other hand, warn that it can potentially lead to reconstruction artifacts due to the increase in activity in the bowel loops^8,10^ and consequently advise early acquisition over late scanning.

## The impact of physical activity

To our knowledge, no studies have examined the role of controlled physical activity while waiting for MPI imaging, and the value of this intervention is uncertain.

Previous research has indicated that walking exercise can significantly influence gastrointestinal motility. For example, Noh *et al*. and colleagues found that intensive walking (exceeding 3000 steps) during bowel preparation before a colonoscopy led to notably higher bowel cleansing scores.^25^ Others have shown that physical activity accelerates colonic transit times.^26,27,28^ For patients with gynaecological cancer, engaging in pre-operative walking was connected to a more rapid recovery of bowel function post-surgery.^29^ The literature also indicates that aerobic exercise can enhance intestinal motility, especially when practiced consistently over several weeks.^30^ However, the mechanisms by which walking stimulates intestinal motility remain unclear.

Consistent with previous literature findings, we assume that adopting a similar approach might reduce artifacts by accelerating gastrointestinal peristalsis after a pharmacological stress test. This could result in a faster clearance of radiopharmaceuticals from the gastrointestinal tract, potentially improving the diagnostic accuracy of the study.

Our recent unpublished data from a randomized study show that the use of electronic pedometer watches encouraged patients to walk while waiting for imaging. However, the number of steps did not affect the occurrence or intensity of gastrointestinal activity-related artifacts, nor did it impact the acceptance rate of scans after pharmacological stress, compared to self-paced walking.

## Conclusions

The problem of subdiaphragmatic activity in MPI is significant, and various approaches have been tested to reduce artifacts, but none have proven effective enough to be included in the guidelines. Based on our experience, some possible approaches to reducing artifacts include choosing stress testing with an exercise stress test, when possible, late imaging, fluid intake, and consuming carbonated water immediately before imaging.
